# Genistein improves mitochondrial function and inflammatory in rats with diabetic nephropathy via inhibiting MAPK/NF-κB pathway

**DOI:** 10.1590/acb370601

**Published:** 2022-08-15

**Authors:** Ying Li, Santao Ou, Qi Liu, Linwang Gan, Liling Zhang, Yujie Wang, Jianhua Qin, Jin Liu, Weihua Wu

**Affiliations:** 1 MD. SiChuan Clinical Research Center for Nephropathy – Affiliated Hospital of Southwest Medical University – Department of Nephrology – Luzhou, China.

**Keywords:** Genistein, Diabetic Nephropathies, Inflammation, Mitogen-Activated Protein Kinases, Genes, p53

## Abstract

**Purpose:**

To investigate the effect of genistein on inflammation and mitochondrial function of diabetic nephropathy.

**Methods:**

Diabetic nephropathy model was established in Sprague-Dawley rats. Automatic biochemical analyzer was employed to detect the kidney function index, serum creatinine, serum urea nitrogen, and 24 h-urine protein and blood glucose. Hematoxylin and eosin staining and periodic acid Schiff staining were used to observe renal morphology. Mitochondrial changes and podocyte integrity were monitored by transmission electron microscope. The expression levels of mfn2, NOX4, P53, MAPK, and NF-κB were detected by Western blotting. The changes of mitochondrial membrane potential were measured by JC-1. The level of mfn2 was assessed by immunofluorescence assay.

**Results:**

Genistein ameliorated the kidney function with reduced Scr and blood glucose. The expressions of NOX4, MAPK, p65 and p53 were downregulated, while the expression of mnf2 was the opposite in genistein-treated kidneys. Further investigations revealed that genistein reduced expansion of mesangial matrix and oxidative stress, protected podocyte integrity and increased mitochondrial membrane potential.

**Conclusions:**

Genistein could alleviate diabetic nephropathy through inhibiting MAPK/NF-κB pathway, improving mitochondrial function and anti-inflammatory.

## Introduction

Diabetic nephropathy (DN) is a microvascular complication of diabetes mellitus which is characterized by high incidence, low diagnosis rate, long duration of illness, high treatment cost, and high mortality^1^. Glucose and lipid metabolism disorders are crucial clinical manifestation of DN^2^. Glomerular lesions are the most momentous pathological changes in clinical DN patients^3^, particularly diffuse and nodular mesangial expansion, and glomerular basement membrane thickening^4^. Studies have shown that many factors play a vital role in the pathophysiological development of DN, including poor glycemic control, hyperlipidemia, oxidative stress, accumulation of advanced glycation end products, and epigenetic factors^5^. However, the pathogenic mechanisms of diabetic nephropathy have not been determined. A large number of data have indicated that the oxidative stress and chronic inflammation play a key role in DN^6^. Although DN’s blood glucose, lipids and blood pressure are strictly controlled, there is lack of effective treatments other than kidney replacement therapy when the course of disease progresses to end-stage renal disease^7^^,^ 8. Hence, it is urgent to find new medicines to treat DN.

It is well known that genistein (GEN) has a variety of molecular activities, including estrogen-like effects, promotion of lipid and glucose metabolism anti-lipid peroxidation^9^^,^ 10, anti-inflammation and anti-fibrosis^11^. It can reduce fasting blood glucose levels in T1D rats and DB/DB mice induced by streptozotocin^12^^,^ 13. So, genistein is appropriate for prevention and treatment of diabetes mellitus. Studies found that high-dose genistein can alleviate mesangial cell lesions of kidney in DM patients^14^. In primary cortical neurons treated with H_2_O_2_, low-dose genistein regulates NF-κB and inhibits apoptosis^15^, and the similar effect is observed in mouse and rat models of ischemia^16^^,^ 17. Moreover, genistein also suppresses NF-κB activation in brain microvascular endothelial cells of rats^18^. These results suggest that NF-κB may be a primary target of genistein, but the exact mechanism of this effect is not clear^19^. In addition, genistein has been shown to activate downstream kinase pathways of growth factors, for instance, PI3/AKT, p38 MAPK, ERK1/2, MAPK, and JNK.

Mitochondrial dysfunction is a central factor in the development of DN. Mfn2 is a dynamic regulatory protein in mitochondria that maintains mitochondrial morphology and biological functions by regulating various signaling pathways (*e.g.*, Ras/MAPK, PERK, Akt/Bax). Mfn2 can inhibit mitochondrial morphological and functional abnormalities by reducing oxidative stress, endoplasmic reticulum stress and autophagy, and ultimately delay the development of DN, so it may be a potential target for DN therapy. However, the specific mechanism of mfn2 in DN remains unclear^20^.

Hence, in the study, the effect of genistein on inflammation based on MAPK/NF-κB pathway and mitochondrial function based on the expression of Mfn2 protein were investigated in DN. This will lay the foundation for the treatment of DN for the time to come.

## Methods

### Animals and group

Male Sprague-Dawley rats (aged 2-3 months and weighed 150 ± 20 g) were purchased from Chengdu Dashuo Biological Co., Ltd (Sichuan, China). The rats were placed in an isolated environment with temperature of 22-24°C and humidity of 40-60% in a 12 h light/dark cycle and were provided with clean drinking water and chow. Five rats were used as control group and 15 for modeling. In this study, all animal experiments were conducted according to the ethical standards of experimental animals (Ethics Committee of Traditional Chinese Medicine Hospital Affiliated to Southwest Medical University).

### Diabetic nephropathy modeling

High-fat and high-sugar diet and streptozotocin were used to establish a DN model^21^. In brief, the rats were fed high-fat and high-sugar diet for eight weeks. Then, after 12 h of fasting without water, a single intraperitoneal injection of 50 mg/kgof streptozotocin (dissolved in sodium citrate buffer, 0.1 M, pH 4.4) was injected into model group. Three days later, the fasting blood glucose level was measured through tail vein blood. It was considered a successful model of diabetic rats when the glucose value of fasting blood was ≥ 16.7 mmol/L. After three weeks, the real-time detection of 24 h proteinuria was performed on these models, in which it was considered as DN model when the 24 h proteinuria was more than 30 mg. The control group was injected with an equal volume of sodium citrate buffer at the same time. DN model rats were randomly divided into two groups: low-dose genistein group (30 mg/kg/d, gavage for six weeks) and high-dose genistein group (50 mg/kg/d, gavage for six weeks) (n = 5).

### Biochemical analysis of blood

Automatic biochemical analyzer was employed to detect the renal function index, serum creatinine, serum urea nitrogen, and serum glucose levels such as glucose.

### Renal pathological changes analysis

The kidney tissues were first fixed in 10% formalin and then embedded in paraffin. Hematoxylin-eosin (H&E) and periodic acid Schiff (PAS) were executed on 4-mm thick paraffin sections to see the morphological alterations of kidney during diabetic nephropathy.

### Western blot

Western blotting was performed as previously reported. Proteins were prepared using radioimmunoprecipitation assay (RIPA) lysis buffer (Abcam, Ab156034, United Kingdom). Protein concentration was determined by Pierce™ BCA protein assay kit (Thermo Fisher Scientific, United States of America). Protein extracts were separated by SDS-PAGE and transferred onto polyvinylidene fluoride membranes. After blocking with 5% non-fat dry milk in Tris-buffered saline, membranes were incubated at 4°C overnight with primary antibodies against mfn2 (ab205236; 1:1,000; Abcam; United States of America), NOX4 (ab 133303; 1:1000; Abcam; United States of America), p53 (ab26; 1:250; Abcam; United States of America), p38 MAPK (ab4822; 1:1000; Abcam; United States of America), and p65 (ab16502; 1:1000; Abcam; United States of America). Subsequently, a secondary goat anti-rabbit IgG (HRP) antibody (Abcam, ab205718, 1:2,000) was applied to incubate with membranes at room temperature for 1 h. The blots were assayed by using the Novex™ ECL chemiluminescent substrate reagent kit (Thermo Fisher Scientific™, WP20005, United States of America). β-actin was used as an internal reference protein. The results were analyzed with ImageJ software.

### Immunofluorescence

As previously mentioned, renal paraffin sections were sealed with 5% BSA for 1 h. Then, the sections were incubated with first antibody mfn2 (sc-sc-515647, 1:200, Santa Cruz) at 4°C overnight. Later, the sections were incubated with secondary antibody at 37 °C for 2 h, which were stained with DAPI for 5-7 min. Finally, the expression of mfn2 was observed by laser confocal microscopy and recorded by taking pictures.

### Transmission electron microscope

As mentioned before, kidney samples were prepared^22^ and fixed in cacodylate buffer (containing 2.5% glutaraldehyde and 2.5% paraformaldehyde, 0.1 M, pH 7.4) for 24 h. The samples were soaked in 1% osmium tetraoxide for 1 h at 4°C and then graded dehydrated with absolute ethanol. Samples were embedded in Epon 812 longitudinally, then cut into ultrathin sections at 70 nm, and compared with uranyl acetate and lead citrate. Finally, the differences were observed by transmission electron microscope at 80 kV.

### JC-1 assay

Mitochondrial potential was measured by JC-1 method. Renal tissues were homogenized in 50 mM ice-cold phosphate buffer (containing 0.1 mM EDTA, pH 7.4) and centrifuged at 2,000 rpm for 10 minutes at 4⁰C. Then, supernatant was collected and again centrifuged at 15,000 rpm for 40 minutes (4°C), and after collection of supernatant occurred. The supernatant (2 × 10/mL) were incubated with 10 μg/mL JC-1 dye (20 min, 37°C, dark). Fluorescence was generated on an Axio Imager M2 microscope (Carl Zeiss), and green JC-1 monomers (Ex/En 490/529, loss of AYm) and orange JC-1 aggregates (Ex/Em 514/590, normal AYm) were viewed simultaneously. The mitochondria uncoupler carbonyl cyanine p-(trifluoromethoxy) phenylhydrazone (FCCP) served as the negative control.

### Statistical analysis

The experimental data were statistically analyzed with Statistical Package for the Social Sciences (SPSS) 17.0 statistical software and GraphPad Prism 7 software. All the data were expressed as mean ± standard error (SD). One-way analysis of variance (ANOVA) was used for differences among the groups. When differences were homogeneous, least significant difference test was used. When differences were heterogeneous, Dunnett’s test was used. P < 0.05 was considered statistically significant.

## Results

### Effect of genistein on blood glucose and renal function in diabetic nephropathy rats

**Figure 1:**
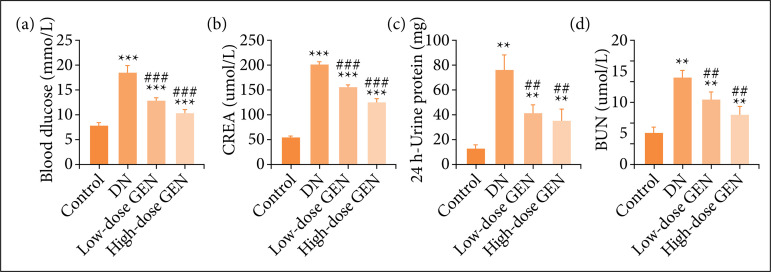
Effect of genistein and blood glucose and renal function in diabetic nephropathy (DN) rats. **(a)** Fasting blood glucose. **(b)** Serum creatinine (CREA). **(c)** 24 h-urine protein. **(d)** Serumurea nitrogen (BUN). Values are indicated as mean ± standard deviation, n = 5 per group.

In order to evaluate the effects of genistein on glucose metabolism and renal function in DN rats, fasting blood glucose, serum creatinine, serum urea nitrogen, and 24 h-urine protein were measured. The levels of fasting blood glucose, serum creatinine, serum urea nitrogen, and 24 h-urine protein in DN group were significantly higher than in control group. After treatment with different concentrations of allicin, their levels decreased significantly (Figs. 1a-1d). Collectively, genistein regulated glucose metabolism and protected renal function in DN rats.

**p < 0.01 *vs*. control group; ***p < 0.001 *vs*. control group;

### Genistein prevented renal pathological lesions in diabetic nephropathy rats

**Figure 2:**
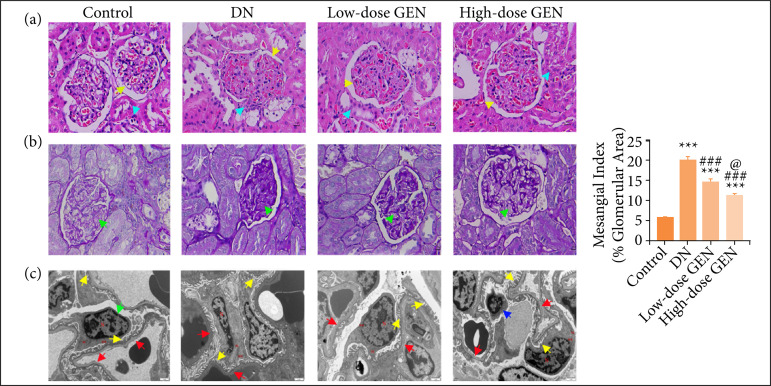
Genistein (GEN) prevented renal pathological lesions in diabetic nephropathy (DN) rats. **(a)** Hematoxylin and eosin staining. Yellow arrow: kidney glomerular basement membrane thickening; blue arrow: inflammatory infiltration of the renal interstitium. **(b)** PAS staining. Green arrow: mesangial matrix expansion in the glomeruli. **(c)** Transmission electron microscope (TEM) of the kidney in different groups (×12,000). Green arrow: podocyte; red arrow: endothelial window pores; yellow arrow: foot processes; blue arrow: mitochondria slightly swollen.

H&E staining, PAS staining, and transmission electron microscope (TEM) were employed to assess the pathological lesion changes in the kidneys, including the glomeruli and podocyte. H&E staining results showed inflammatory infiltration of the renal interstitium, glomerular atrophy and degeneration of renal tubule, and thickening of kidney glomerular basement membrane in DN rats (Fig. 2a). PAS staining indicated that mesangial matrix expansion in the glomeruli was observed in DN rats (Fig. 2b). Overtly, genistein could appropriately alleviate these pathological variations, indicating that genistein delayed the progression of DN by alleviating the increased renal lesions (Figs. 2a and 2b).

***p < 0.001 *vs*. control group;

In order to further verify the above phenomenon, we also observed podocyte ultra-structures by TEM. Result revealed that glomerular filtration barrier and tubular morphological structure showed obvious abnormalities: thylakoid cells and vascular endothelial cells were proliferated, endothelial window pores appeared large fusion, the basal membrane of podocyte was thickened and obvious foot processes, most mitochondria in the cytoplasm were swollen, and a large number of rough endoplasmic reticulum had expanded in a cystic shape. A small amount of autophagosomes and lipid droplets were also seen in DN rats. Genistein treatment reduced podocyte damage (Fig. 2c).

### Effect of genistein on mitochondria in diabetic nephropathy rats

**Figure 3:**
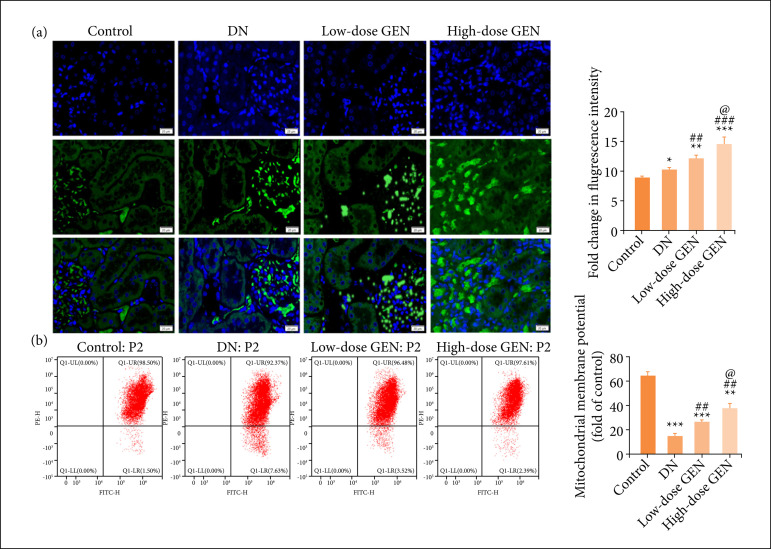
Effect of genistein (GEN) on mitochondria in diabetic nephropathy (DN) rats. **(a)** Immunofluorescence staining for mfn2 (20 μm, ×400 magnification). Green fluorescence: the expression of mfn2. Blue fluorescence: the nucleus reinfected with DAPI. The bar chart shows the quantitative analysis of fluorescence intensity. **(b)** JC-1 for mitochondrial membrane potential. Values are expressed as mean ± SD, n = 5 per group.

Mitochondrial dysfunction is an important factor for pathogenesis of DN. Mfn2 is a multifunctional protein, which promotes mitochondrial fusion, and it is essential to maintain the normal function of mitochondria. To uncover the underlying molecular mechanisms of genistein on renal protection in diabetic nephropathy, we analyzed changes of mitochondrial membrane potential and assessed the level of mfn2. The mfn2 expression and mitochondrial membrane potential in DN group were significantly higher than those in control group, which were also increased in each treatment group with genistein in a dose-dependent manner (Figs. 3a and 3b). It shows that genistein supplementation improves nephropathy likely via modulation of the mitochondrial fusion and function in DN rats.

*p < 0.05 *vs*. control group; **p < 0.01 *vs*. control group; ***p < 0.001 *vs*. control group;

### Effect of genistein on NF-κB/ MAPK/p53 pathway in diabetic nephropathy rats

**Figure 4:**
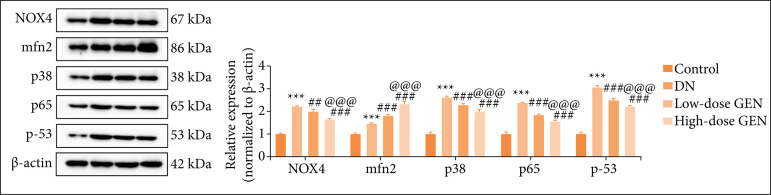
Effect of genistein (GEN) on NF-κB/MAPK/p53 pathway in diabetic nephropathy (DN) rats. The expressions of NOX4, mfn2, p38 MAPK, p65, and p53 in each group were detected by Western blot. Values are expressed as mean ± standard deviation, n = 5 per group.

There is sufficient evidence that oxidative stress and inflammation play a key role in the progression of DN. NOX family may be the main source of reactive oxygen species (ROS) in kidney cells of DN and mediate the occurrence of oxidative stress. In addition, MAPK pathway regulates inflammation process. Moreover, NF-κB promotes the release of proinflammatory cytokines in renal tissue. The expression of NOX4, mfn2, p38 MAPK, p65, and p53 were assessed by Western blots to investigate the effect of genistein on NF-κB/MAPK/p53 pathway. Compared with control group, NOX4, mfn2, p38 MAPK, p-65, and p-53 levels of DN group were notably decreased. Compared with DN group, NOX4, p38 MAPK, p65, and p-53 levels of genistein group were significantly reduced in a dose-dependent manner, but mfn2 expression was the opposite. Thus, there was attenuation of oxidative stress and inflammation, enhancement of mitochondrial fusion in renal tissue of DN rats that were treated with genistein (Table 1, Fig. 4).

**Table 1:** Protein relative expression levels (x ± standard deviation, *p*)*.

	Control group	DN group	Low-dose GEN group	High-dose GEN group
NOX4	1.00 ± 0.05	2.21 ± 0.04***, 0.000	2.00 ± 0.08##, 0.001	1.67 ± 0.02### @@@, 0.000
mfn2	1.00 ± 0.04	1.48 ± 0.03***, 0.000	1.82 ± 0.04###, 0.000	2.33 ± 0.08### @@@, 0.000
p38	1.00 ± 0.08	2.63 ± 0.04***, 0.000	2.29 ± 0.07###, 0.000	2.00 ± 0.08### @@@, 0.000
p65	1.00 ± 0.07	2.39 ± 0.01***, 0.000	1.86 ± 0.03###, 0.000	1.57 ± 0.03### @@@, 0.000
p53	0.99 ± 0.06	3.08 ± 0.04***, 0.000	2.52 ± 0.04###, 0.000	2.21 ± 0.80### @@@, 0.000

DN: diabetic nephropathy; GEN: genistein;

* values are expressed as mean ± standard deviation, n = 5 per group;

*** p < 0.001 *vs*. control group;

## p < 0.01 DN group;

### p < 0.001 DN group;

@@@ p < 0.001 *vs*. low-dose GEN group.

***p < 0.001 *vs*. control group;

## Discussion

In the world, one of the main causes of end-stage renal failure is DN^23^. Interstitial fibrosis, tubular degeneration, and glomerulosclerosis are typical pathological changes of DN, which can lead to end-stage renal failure^24^^,^ 25. The treatment of DN is more complex than that of general kidney diseases, and its prognosis is weak. At present, the understanding of the pathogenesis of DN is insufficient, and there is neither effective prevention nor treatment measure. Genistein is a kind of chemical substance extracted from soybean, which has been proved to be an antioxidant, inflammation regulator, promoting mitochondrial protection and immunosuppressive effect in clinic^19^. Particularly, genistein has been certified to regulate some pathophysiologic pathways, which usually play a role in cancer, metabolic syndrome, and obesity^26^. Therefore, we studied the possible mechanism of genistein protecting the kidney of DN. In the study, the data showed that genistein attenuates DN by inhibiting MAPK/NF-κB pathway and improving mitochondrial function.

In this study, Sprague-Dawley rats were injected intraperitoneally with streptozotocin to establish a DN model. The results showed that genistein could decrease the renal histopathological damage by reducing the inflammatory infiltration and glomerular basement membrane thickening, and inhibiting the expansion of mesangial matrix and podocyte autophagy in DN rats. In addition, genistein could significantly decrease Scr and blood glucose levels, which indicated genistein could regulate glucose metabolism and protect renal function in DN rats. In conclusion, genistein may be a new medicine in the treatment of DN. This antioxidant function is mediated by the mitochondrial function of genistein.

Mfn2 is a multifunctional protein, which promotes mitochondrial fusion, regulates mitochondrial transport and keeps mitochondrial DNA stability. Both basic and clinical studies show that Mfn2 plays an important role in biogenesis and metabolism in diabetes. Some studies have shown that appropriate mfn2 expression in mitochondria is helpful to maintain normal mitochondrial morphological integrity and stability^20^. The degree of mitochondrial division in patients with DN increased with the decrease of Mfn2 protein level. Liang *et al*.^27^ detected after global cerebral ischemia 1 mg/kg genistein (intravenous injection, 5 minutes) can decrease the production of mitochondrial ROS and promote the release of cytochrome C activated by caspase, thereby alleviating hippocampal injury by activating antioxidant pathway. This antioxidant function is mediated by the mitochondrial function provided by genistein^28^. In the study, genistein enhanced mitochondrial membrane potential and promoted Mfn2 protein expression in a dose-dependent manner. Therefore, genistein slowed the progression of DN to improve mitochondrial function.

Oxidative stress and inflammation are closely related in DN^29^. Recent studies revealed that one of the main sources of ROS production is renal cells in DN patients^30^. It has been reported that ROS plays a significant role in the pathogenesis of renal fibrosis, because it could mediate the mechanism of a variety of multiple profibrotic factors in inducing fibroblast proliferation and/or activation^31^. In addition, ROS could also regulate the signal transduction of NF-κB and p38MAPK signaling pathway and promote the release of renal inflammatory cytokines.

The antioxidant properties of genistein have been widely used in various researches^32^. In studies with 18 macrophages, genistein blocked oxidative stress induced NF-κB activation and inhibited gene expression related to immune inflammation. In addition, genistein inhibits the migration of inflammatory cells by reducing the adhesion of leukocytes to endothelial cells^33^. Kindy^34^ showed that 30 minutes preconditioning or 5 minutes postconditioning could reduce the loss of hippocampal neurons induced by transient global cerebral ischemia in male gerbils. This therapeutic effect is achieved by inhibiting MAPK phosphorylation and further inhibiting MAPK pathway^34^. In the study, results showed that genistein up-regulated NOX4, MAPK, p-65, and p-53, which indicated genistein suppressed NF-κB/MAPK/p53 pathway. Accordingly, genistein controlled inflammatory reactions and oxidative stress.

## Conclusion

Genistein protected mitochondria by up regulating mfn2 expression to delay DN progression. Furthermore, genistein also improved renal injury on account of DN via inhibiting oxidative stress and inflammatory reactions and via restraining NF-κB/p53 MAPK pathway.
